# Less fit *Lamium amplexicaule* plants produce more dispersible seeds

**DOI:** 10.1038/s41598-019-42158-1

**Published:** 2019-04-19

**Authors:** Eyal Zinger, Ariel Gueijman, Uri Obolski, Yoav Ram, Eliya Ruby, Mor Binder, Nivi Yechieli, Nir Ohad, Lilach Hadany

**Affiliations:** 1 School of Plant Sciences and Food Security, Tel Aviv University, Tel Aviv, Israel; 2 School of Public Health; Porter School of the Environment and Earth Sciences, Tel Aviv University, Tel Aviv, Israel; 3 School of Computer Science, IDC Herzliya, Herzliya, Israel

**Keywords:** Behavioural ecology, Evolutionary ecology, Coevolution, Natural variation in plants

## Abstract

Theory predicts that less fit individuals would disperse more often than fitter ones (Fitness Associated Dispersal, FAD hypothesis). To test this prediction under laboratory conditions, an entire life cycle of *Lamium amplexicaule* plants and the preferences of its dispersal agent, *Messor ebeninus* ants, were tracked. Characterization of individual *L. amplexicaule* plant revealed high variability in spot cover on the surface of the seeds, where less fit plants produce “unspotted seeds” (see Fig. 1 in Introduction). Unspotted *L. amplexicaule* seeds showed higher variation in germination time and lower germination rate. Moreover, *M. ebeninus* ants preferably collected these unspotted seeds. Our results show that low fitness *L. amplexicaule* plants produce seeds with higher potential for dispersal, supporting the FAD hypothesis in a plant-animal system.

## Introduction

Dispersal, the spreading of organisms from one place to another, is one of the major forces shaping ecology and evolution. It allows species to conquer various and diverse habitats^1^, avoid harmful interactions within as well as between species^2^ and it may affect gene flow and genetic diversity^3^. Dispersal also carries significant costs, that range from the time and energy^4^ it requires, through increased exposure to predation^5^, and eventually to the risk of not finding a suitable site for settling and reproduction^6^. When considering plants, dispersal may also impair germination and propagation^7^. Furthermore, a plant’s mere survival in a given patch is evidence that the patch allows the growth of that plant^8^. Therefore, dispersal is often considered to cause a reduction in fitness, known as “migration load”^9^, due to the movement of a certain genotype to a novel and untested patch. However, the “migration load” might be mitigated when the home patch is crowded^10–12^, or when dispersal is directed to specific patches, such as in the case of zoochory, dispersal by animals. In some cases, dispersal by animals also promotes germination success^13^.

Theoretical models predict that Fitness Associated Dispersal (FAD) – where the less fit individuals within the population disperse themselves or their offspring more often than the fitter ones^14,15^ – is likely to evolve, as it increases the likelihood of less-successful genotypes to move to a different environment or to outcross with a different genotype^16^.

Similar phenomena have been studied under different names, including genotype-dependent dispersal^17,18^, dispersal plasticity^19^, habitat selection^20^, conditional movement^21^, directed movement^22^, and condition-dependent dispersal^9,23^. Furthermore, such a dispersal pattern can benefit the population as a whole in the long term, including an increased ability for complex adaptation^14^, and a better match between a given genotype and its environment^9^.

While reports describing FAD in plants are limited^24^, several experimental studies showed that less fit animals disperse more often than their successful group members^19,24,25^. This form of dispersal might be explained by different mechanisms, including: (i) actively driving weak group members away by stronger ones^26–33^, (ii) rejection of less fit individuals by potential mates^27,33,34^, and (iii) the FAD hypothesis. Plants systems offer an opportunity to separate the different explanations, as plants are less affected by (i) and (ii). We sought to test the predictions of the FAD hypothesis, specifically in the context of animal-based seed dispersal.

Sessile organisms such as plants often experience changes in their environment and cannot avoid these changes by moving to a different location within the same generation. The ability of plants to move their offspring to a different habitat is crucial for their survival and is mostly dependent on their ability to disperse their genetic content in the next generation via pollen and seed dispersal.

**Figure 1:**
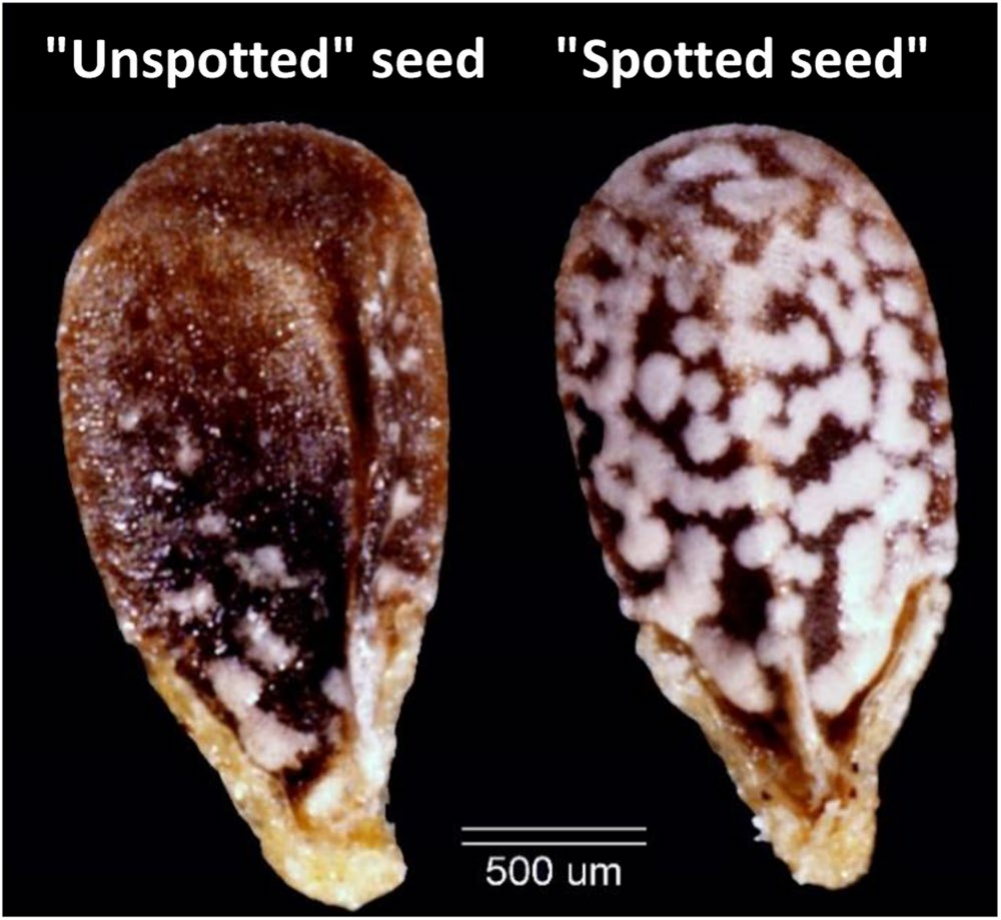
*L. amplexicaule* phenotypes of seed spot cover. We term the least spotted 15% of the seed population as “unspotted” (left) and the top 15% as “spotted” (right).

In this study we tested seed dispersal by *Lamium amplexicaule*, an annual herb dispersed by ants. The seeds were characterized with a focus on the spot cover (see Fig. 1) on the surface of a seed, which is highly variable in *L. amplexicaule*. Furthermore, we tested the effect of seed spot cover on the myrmecochorous interaction between *L. amplexicaule* and one of the its major natural dispersers– the ant *Messor ebeninus*.

*M. ebeninus* plays a major role in dispersing elaiosome-bearing seeds including *L. amplexicaule* and was previously documented shaping plants germination and density by preferential seed dispersal^35^. Following a myrmecochorous interaction, a plant such as *L. amplexicaule* may benefit from the dispersal of its seeds, which may also experience increased germination rate and productivity^36,37^.The elaiosomes of seeds are nutritious for the ants larvae^38,39^ and the ants often eat the elaiosome and leave the seed unharmed^40,41^.

Based on the FAD hypothesis we predict that less fit *L. amplexicaule* plants would produce seeds that are preferred by their dispersers, the ants. Furthermore, we predict that less fit plants would produce seeds that are more variable in their germination. To test these predictions, we characterized plant and seed phenotypes, including plant fitness, seed morphology, germination, and ant preference. We found that seeds with low levels of spot cover (‘unspotted’) were more commonly produced by low fitness mother plants. Unspotted seeds were preferred over spotted seeds by one of the natural dispersers – *M. ebeninus*. Altogether, our results suggest that less fit mother plants can produce seeds with a higher probability of dispersal, supporting the FAD hypothesis.

## Results

### Seed phenotype variation in a population

**Figure 2:**
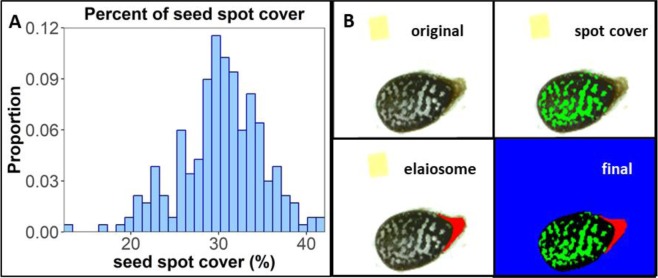
**(A)** Histogram of the percent of seed spot cover. The histogram demonstrates the variability of seed spot cover in a *L. amplexicaule* population. **(B)** An output of SID, a seed image processing tool. On the top left square is the original seed photo, on the top right square is the spot cover layer marked in green. On the bottom part to the left is the elaiosome layer marked in red and to the right is the final output of the image processing tool with all fetched layers. The percent of seed spot cover (green layer, data shown on (**B**) and Fig. 3) was acquired by these measurements (n = 30 plants, 486 seeds).

*L. amplexicaule* plants were grown in two separate samples differing in light intensity regimes (see Methods). First, we found that seed spot cover was highly variable in each of the sampled groups, from 20% to 40% (486 seeds representing one seed per whorl of each of 30 plants sampled, see Fig. 2(A)). Next, we tested the association between seed spot cover and three major measures: plant fitness, germination probability, and ant preference.

### Seed phenotype and plant fitness

The correlation between seed traits and plant fitness was tested on two samples, one grown under low light intensity, and the other under high light intensity (see Methods).

**Figure 3:**
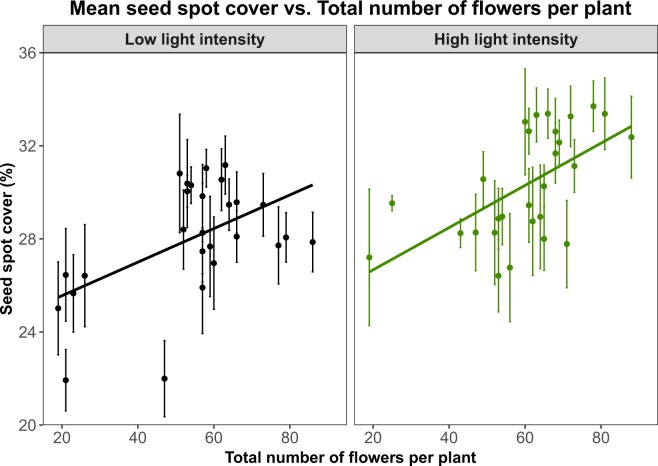
The total number of flowers per plant (reflecting fitness) is positively correlated with mean seed spot cover: plants with lower fitness produce unspotted seeds. The plot shows two independent samples differing in the light intensity used in the growth chamber: black, low light intensity and green, high light intensity. Shown are observations per plant (points) and their respective linear models (lines). Mean seed spot cover percentage per plant and total number of flowers per plant were highly correlated for both samples (low light: t = 3.01, p-value = 5.9 · 10^−3^, *R*^2^ = 0.24; high light: t = 3.02, p-value = 5.5 · 10^−3^, *R*^2^ = 0.24; linear regression, n = 30 plants for both samples). The linear model suggests that plants had unspotted seeds when their fitness was lower.

For each sample we used the total number of flowers in a plant as a proxy for the fitness of the plant (see Measuring plant fitness in the Methods section) and tested its association with the mean spot cover of its seeds. We found that plants with fewer flowers produced more unspotted seeds for both samples (low light: t = 3.01, p-value = 5.9 · 10^−3^, *R*^2^ = 0.24; high light: t = 3.02, p-value = 5.5 · 10^−3^, *R*^2^ = 0.24; linear regression, n = 30 plants for both samples, see Fig. 3 in black and green respectively). Plant total number of flowers was not significantly associated with seed area (low light: t = −1.139, p-value = 0.27, *R*^2^ = 0.01; high light: t = 0.81, p-value = 0.42, *R*^2^ = 0.02; linear regression; see Fig. S4), estimated weight (low light: t = 1.877, p-value = 0.07, *R*^2^ = 0.09; high light: t = 1.113, p-value = 0.28, *R*^2^ = 0.01; linear regression; see Fig. S5), or variance in seed cover (low light: t = 0.283, p-value = 0.78, *R*^2^ = 0.01; high light: t = 0.322, p-value = 0.75, *R*^2^ = 0.01; linear regression, see Fig. S12). However, it was positively correlated with elaiosome size (low light: t = 4.914, p-value = 4.66 · 10^−5^, *R*^2^ = 0.47; high light: t = 2.603, p-value = 0.015, *R*^2^ = 0.18; linear regression; see Fig. S6). At the seed level, seed cover was positively correlated with seed area (low light: t = 3.11, p-value = 2 · 10^−3^; high light: t = −2.84, p-value = 5 · 10^−3^; mixed effect linear regression, see Fig. S7) but not correlated with elaiosome size (low light: t = 1.165, p-value = 0.25; high light: t = −1.421, p-value = 0.16; mixed effect linear regression, see Fig. S8).

The high and low light intensities showed similar slopes (see Fig. 3, 0.062 for high light and 0.078 for low light intensity). However, seed spot cover and seed elaiosome area were higher in the high light intensity (see Fig. S9(a) and (b)), whereas the total number of flowers per plant did not differ significantly between light intensity regimes (see Fig. S9(c)).

### Seed phenotype and Germination

**Figure 4:**
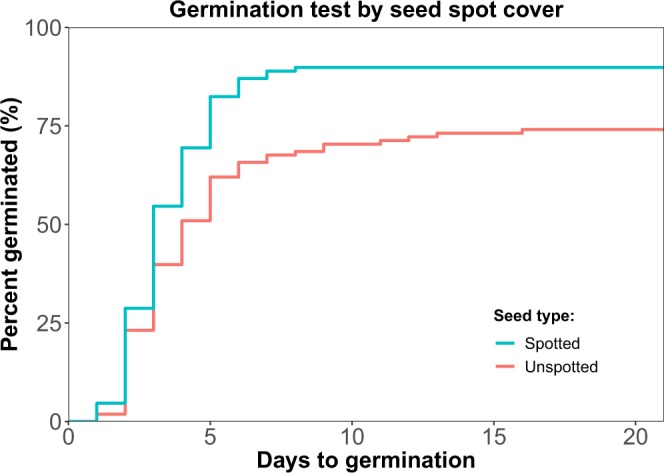
Germination test: spotted seeds have a higher germination rate and lower variation in germination time. An inverse Kaplan-Meier survival plot shows the proportion of spotted (blue) and unspotted (red) seeds germination (y-axis) as function of time in days (x-axis). Unspotted seeds germinated in lower proportion compared to spotted seeds (n = 108 seeds for each category, Wilcoxon rank-sum test, W = 3118, p = 0.024), and their germination time was more variable (*χ*^2^ = 11.2, DF = 1, p-value = 8.14 · 10^−4^, log rank-sum test).

The unspotted seeds germinated at a lower rate (n = 108 seeds for each category, 74% and 89.8% germination for unspotted and spotted seeds, respectively. Wilcoxon rank-sum test, W = 3118, p-value = 0.024) and demonstrated greater variance in germination time (log rank-sum test *χ*^2^ = 11.2, DF = 1, p-value = 8.14 · 10^−4^) compared with spotted seeds (see Fig. 4).

### Ant seed preference

**Figure 5:**
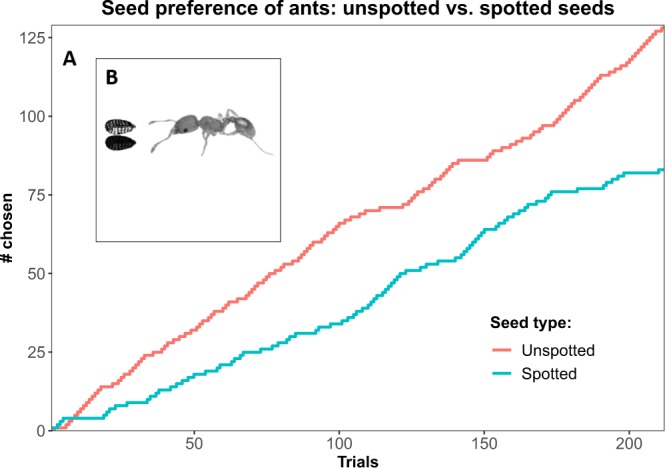
**(A)** The total number of spotted (blue) and unspotted (red) seeds removed by the ants over 212 trials were 83 and 129, respectively. In 60.8% of the trials (n = 212 trials, OR = 0.391, p-value = 0.024), the ants preferred the unspotted seeds. **(B)** The experimental setup in the arena in which pairs of spotted and unspotted seeds were presented to *M. ebeninus* ants (see detailed scheme in Fig. S3).

**Figure 6:**
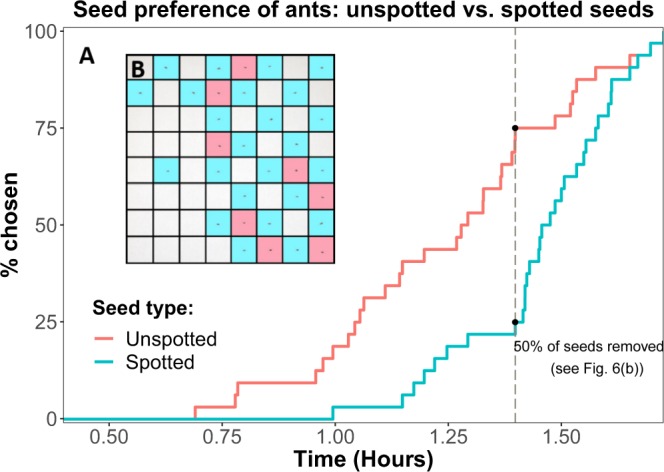
Ants prefer unspotted seeds: Grid-based method. **(A)** An inverse Kaplan-Meier plot of ants’ seed preference in a grid-based experiment, where the Y-axis presents the proportion of seeds removed. Seeds were divided to two groups: spotted seeds (blue) and unspotted seeds (red). The vertical dashed line indicates the time where half of the total seeds were taken (see **B**). The experiment showed that unspotted seeds were removed before spotted seeds (p < 0.0008, n = 32, Wilcoxon rank-sum). **(B)** The grid after 50% of the seeds were removed by ants in an 8-by-8 grid-based seed preference experiment, where unspotted and spotted seeds were arranged in a checkers board format (with the board itself uniformly white). Square color was retrospectively added for visualization, to highlight the type of the remaining seeds on the grid - spotted (blue) vs. unspotted (red). Empty squares stand for collected seeds.

By the trial-based method, Unspotted seeds had significantly higher odds of being removed (n = 212 pairs, p-value = 0.024, mixed-effects logistic regression, OR = 0.391, see Fig. 5). Using the grid-based method, we found that unspotted seeds were removed before spotted ones (p-value = 7.4 · 10^−4^, Wilcoxon rank-sum test, see Fig. 6(A)), suggesting that the ants preferred removing unspotted seeds, consistent with the results of the trial-based test. For example, following the removal of 50% of all seeds, only 8 unspotted seeds remained in contrast to 24 spotted ones (see dashed line in Fig. 6(A) and illustration in Fig. 6(B)).

## Discussion

In this study we show that *L. amplexicaule* seeds display natural variation in seed spot cover, which can be observed even within the context of a single seed lineage (see Fig. 2). Plants with lower fitness tended to have unspotted seeds (see Fig. 3). Unspotted seeds showed both lower rate of germination and higher variance in germination time (see Fig. 4). Finally, we tested the preference of *M. ebeninus* ants, natural dispersers of *L. amplexicaule* seeds, and demonstrated their preference to unspotted seeds in comparison with spotted ones using two independent preference assays (see Figs 5(A) and 6(A) and video link in Supplementary). The observed preference of the ants for unspotted seeds could not be explained by size of the seed (unspotted seeds tended to be smaller than spotted ones, see Fig. S7) or the the size of the elaiosome (no difference was observed between the elaiosomes of spotted and unspotted seeds, see Fig. S8). Altogether, our results support the FAD hypothesis^16^, showing that less fit plants produce seeds which are better suited to be dispersed.

Low seed spot cover was correlated with both lower rates of seed germination and higher ant preference (potentially leading to seed dispersal by the ants). This association can be interpreted in two ways: First, low seed spot cover might be a result of the inability of the less fit mother plant to produce spotted seeds due to limited resources. Second, a less fit mother might be paying a higher cost for increased dispersal by ants, which may be favored when fitness is low, due to either rough environmental conditions or a bad genotype.

Unspotted seeds also showed higher variance in germination time that can be viewed as an adaptation to an ever-changing environment. It is believed that temporal variation in germination and seed dormancy provide bet hedging strategies for plants, especially in the context of annuals^42,43^. The combination of variation in germination time and enhanced dispersal of seeds of less fit plants may increase the probability that some of these seeds would avoid the harmful conditions encountered by the mother-plant (see Fig. S2). However, we do not consider low seed spot cover as the cause of these phenomena but rather that the three phenomena might be associated, possibly through another factor (e.g. energetic limitation). Irrespective of its cause, the association between fitness and dispersal may result in an increased probability for survival and adaptation in future generations.

To consider the evolutionary role of ant-dispersed seeds, we need to consider the survival of the seeds in the ants nest^44–46^. Following the myrmecochorous (i.e. dispersal by ants) interaction, the *L. amplexicaule* seeds are placed in seed storage compartments in the ants’ nest, and it is common to observe patches of *L. amplexicaule* seedlings above the edges of the ants’ nest at the beginning of the rainy season (see Fig. S1). In addition, these compartments protect the seeds from biotic and abiotic factors and enable the seeds to remain relatively dry until the first substantial rain occurs. Furthermore, it was suggested that the mere presence of ants in proximity to *L. amplexicaule* seeds reduces their predation^47^. Interestingly, the *L. amplexicaule* seeds germinate at lower rates on the face of the ground as compared to germination at a depth of 3–6 cm (Table S1), possibly reflecting an adaptation to germination within an ant nest.

We suggest that *L. amplexicaule* plants can adjust the spot cover of their seeds in an adaptive manner and thus regulate their seed dispersibility by ants. The theory of fitness associated dispersal suggests that such an adjustment could be advantageous for two main reasons: first, a less fit plant allows more of its offspring to move to a different location, possibly avoiding an environmental stress. Second, the neighbors of the offspring would more often be of a different genetic background, resulting in a higher likelihood of mixing of different genotypes, potentially facilitating adaptation. Thus, fitness associated dispersal allows movement in the physical and genetic spaces at the same time.

A cost of dispersal, as observed here, may further decrease the chances of less fit genotypes to disperse their seeds. Consequently, deleterious mutations would be eliminated from the population more effectively, potentially resulting in a long-term advantage to the population as a whole^48,49^. This may also result in increased fixation probability of beneficial mutations, and in particular beneficial combinations^14^.

Altogether, our results support the FAD hypothesis and suggest that some animal-dispersed plants could have evolved to change their seed traits - and consequently their dispersibility - according to their fitness. By producing seeds that are favored by the animals dispersing them when the plant is maladapted, the mother plant can regulate the dispersibility of its offspring. Our results imply that similar mechanisms might play a role in the effect of stress on seed phenotype in other animal-dispersed plants^50^, including many crops.

## Methods

To address the relationship between plant fitness, seed properties and dispersibility, we examined the myrmecochorous plant species *Lamium amplexicaule*^51^, and its main seed disperser, the harvester ant *Messor ebeninus*, as a model system. *L. amplexicaule* seeds have yellowish semi-transparent elaiosome attached to them - a lipid and protein rich tissue on the edge of a seed^51,52^. *L. amplexicaule* displays high variation in both its reproductive and dispersal strategies. A single individual may simultaneously present self-fertilizing (cleistogamous) and facultative outcrossing (chasmogamous) flowers^53^, and high variation of seed phenotypes (see Fig. 1), from light colored (“spotted”) to dark (“unspotted”). *L. amplexicaule* seeds were sampled in March (spring) from a natural population in Netzer Sereni (31°55′33.96″N 34°49′46.2″E), Israel. *L. amplexicaule* plants germinate in Israel at the beginning of the rainy season (November or December) and produce seeds from February to April in natural conditions. Plants were cultivated and propagated for three generations, to obtain single seed descent.

### Plants growth conditions

*L. amplexicaule* seeds were germinated and grown in a growth chamber for three consecutive life cycles. Seedlings originating from the general population were used for the first cycle (n = 35 plants). Seedlings originating from a single seed descent were used for the second cycle (n = 35 plants) and the third cycle (n = 51 plants). Plants from all cycles were grown on a standard nutrient-rich soil mix under artificial wide-spectra fluorescent light (Floura, Daylight and Cool White lamps by OSRAM, 50–100 μE for low light intensity regime and 100–200 μE for high light intensity regime) for 16-hours, 24 °C LD (long day) regime and 18 °C at dark. Irrigation with dH_2_O was constantly applied during the entire life cycle of the plants. Seeds were collected from each plant in all cycles for cultivation, seed characterization and ants’ preference assays.

### Measuring plant fitness

The number of flowers for each *L. amplexicaule* plant was monitored through their entire lifecycle, to obtain the total number of flowers, a proxy for the fitness of the plant^54,55^ (see comment 1. in the Supplementary).

Flower count from the top whorl of each plant was omitted since the development of the last whorl was partial and its exclusion was made to avoid over representation of that whorl.

### Characterization of seeds via imaging

The shoot of a *L. amplexicaule* plant is composed of branches carrying flowering whorls; a sample of seeds (n = 486 seeds) was collected from each whorl of the central branch originating from single-seed descent plants (n = 60 plants). Seeds were photographed for characterization using a USB microscope (500X magnitude, resolution 2.0 MP).

The acquired photos were analyzed using an in-house developed tool called *Sid*^56^ (http://sid.readthedocs.io), implemented using Python^57^, NumPy^58^, SciPy^59^, Scikit-image^60^ and Matplotlib^61^. *Sid* identifies the seed, the seed spot cover, and the elaiosome, and measures their shapes (see Fig. 2(B)). Seed spot cover was calculated for each seed by dividing the measured area of the seed spot cover layer (green layer in Fig. 2(B)) by the total area of the seed (the inverse of the blue layer in Fig. 2(B)) to obtain the percent of spot cover (see Figs 2(A) and 3). For each plant we collected 1 seed per flowering whorl, calculated the mean percent of spot cover for the seeds of the entire plant, and counted the total number of flowers (as a measure of fitness). Low level of seed spot cover was defined as the bottom 15 percent of the seed spot cover distribution and was referred to as “unspotted” seeds, and the top 15 percent of the distribution was referred to as the “spotted” ones.

### Ants’ preferences experiments

*M. ebeninus* ants were identified as major natural dispersers of *L. amplexicaule* seeds (in field observations, see Fig. 2S). Thus, an ant colony from Weizmann institute’s natural lands (31.9075° N, 34.8092° E) was brought to Tel Aviv University (courtesy of prof. Feinerman’s lab, Weizmann institute of science, Israel) and was kept in a rearing room under a controlled temperature of 24 ± 2 °C and a photoperiod of 16 L:8D. The nest of the colony was connected through plastic tube (1 cm in diameter and 40 cm in length, kept open throughout the experiments) to a square experimental arena (1 *m*^2^; see Fig. 3S), where seeds from two extreme categories with respect to spot cover were introduced to the ants (see Figs 5(A) and 6(A)). The experimental arena was open to the ant colony, and there were cases where multiple ants took part in the experiments simultaneously. The ants’ preference was tested using two methods:

#### Trial-based method

seeds from the two distinct spot cover categories were randomly assigned to pairs and placed next to each other in the experimental arena (see Fig. 5(B)). When one of the seeds was removed by an ant, the other was removed from the arena by the experimenters. At the end of the session, the number of removals of each condition was divided by the total number of trials to obtain an estimate for the probability of a seed being removed for each category. All together there were 212 valid trials in three days of experiment (Jan 21, 12:00–18:00 between 15:00 and 16:20, n = 30; Jan 23 between 12:00 and 13:00, n = 15; Jan 24 between 15:00 and 18:00, n = 167). The time of day had no significant effect.

#### Grid-based method

Seeds of the two distinct categories were placed on an 8 × 8 grid in a chess board pattern (where the immediate neighbors of each seed are of the other category). To track the foraging pattern of the ants, the arena was continuously filmed using Logitech web camera and iSpy software (by ‘Developer in a box’) for the entire duration of the experiment, September 8–9, and the ants were observed foraging during 23:40–01:30. The output was analyzed with respect to the time and order of the removal (see Fig. 6 and example video link in Supplementary).

### Statistical analysis

The statistical analysis was performed using R^62–65^. The differences in seed spot cover characteristics were analyzed using a linear regression model. Germination time was analyzed using Wilcoxon Rank-Sum test, as the distribution of the data was non-normal and thus required non-parametric analysis. The Trial-based experiments were analyzed using logistic mixed-effect regression, the results from three days of experiments were merged for statistical analysis; a random effect intercept was added to each time point, to account for the interdependencies (n = 212 trials for all three days of experiment). For the analysis of the grid-based experiment, the removal order of seeds from different phenotypes was monitored and compared using the non-parametric Wilcoxon rank-sum test.

## Supplementary information

Supporting Information

Ants grid experiment -supporting information
