# Supplementary material for: Less fit Lamium amplexicaule plants produce more dispersible seeds
Source: Sci Rep. 2019 Apr 19;9:6299. doi: 10.1038/s41598-019-42158-1 (PMC6474898; doi:10.1038/s41598-019-42158-1)
Supplement: Supplementary file 1 — Supporting Information [file 41598_2019_42158_MOESM1_ESM.pdf]

Less fit *Lamium amplexicaule* plants produce more dispersible seeds.

Eyal Zinger[1], Ariel Gueijman[1], Uri Obolski[1,2], Yoav Ram[1,3], Eliya Ruby[1], Mor Binder[1], Nivi Yechieli[1], Nir Ohad[1], and Lilach Hadany[1,*]

[1]Molecular Biology and Ecology of Plants, Tel Aviv University, Tel Aviv, Israel

[2]University of Oxford, Department of Zoology, Oxford, UK

[3]Department of Biology, Stanford University, Stanford, United States

[*]lilach.hadany@gmail.com

Supporting Information

Fitness evaluation methods and additional analysis

Two possible measures of plant fitness were considered: number of flowers [54,55], and number of seeds [54,55]. While the number of seeds is the more common measure, we noticed that it is significantly biased with respect to *L. amplexicaule* plants growing in the lab, in the absence of pollinators. Specifically, chasmogamous flowers (i.e. capable of outcrossing) produced fewer seeds than self-pollinating flowers in the lab in the absence of pollinators (usually 2-3 seeds for a chasmogamous flower in comparison with 4 seeds for a cleistogamous one). However, this is not necessarily the case in the field. Number of flowers measure was thus used since we expect it to be better correlated with the potential amount of seeds an *L. amplexicaule* plant produces outdoors.

In low light regime all but one plant produced only cleistogamous flowers, whereas most plants in high light regime had chasmogamous flowers (ranging between 0% and 30%), a response that was previously documented[53]. Overall, chasmogamy was positively correlated with the number of flowers per plant in high light intensity regime (t= 2.53, p-value =0.018, R^2= 0.17; linear regression). Chasmogamy also showed an increasing trend with the total seed weight (t= 2.02, p-value =0.054, R^2= 0.11; linear regression, see Fig. S10) but we suspect this is an underestimation of the association due to reduced seed production of chasmogamous flowers in the lab.

Furthermore, plants total number of flowers was not significantly correlated with seed area (Fig. S4), estimated mean seed weight (Fig. S5), but was positively correlated with elaiosome size (see Fig. S6). Plants with higher proportions of chasmogamous flowers also had seeds with higher seed spot cover (t= 2.402, p-value =0.025, R^2= 0.15; linear regression, see Fig. S11) which may suggest a trade-off between seed and pollen dispersal.

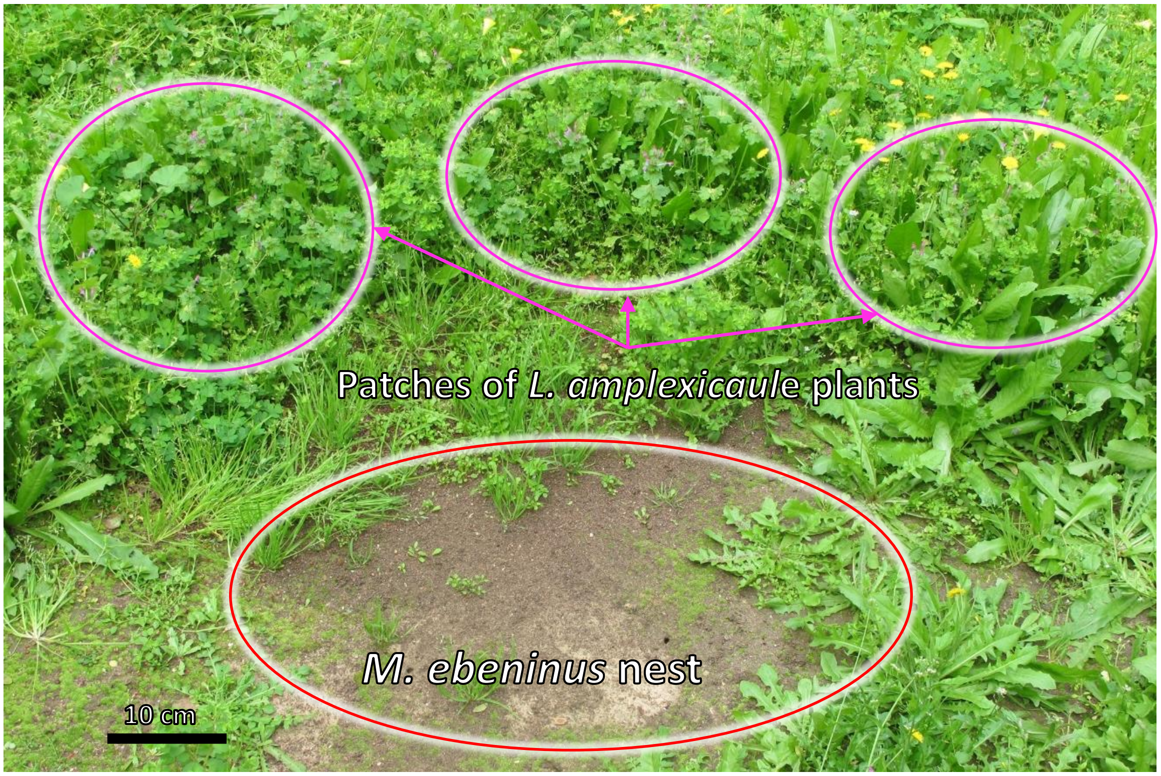

Figure S1 An *M. ebeninus* nest (the main oval that is mostly clear of any plants) and three adjacent patches of *L. amplexicaule* plants in the outskirts of the nest.

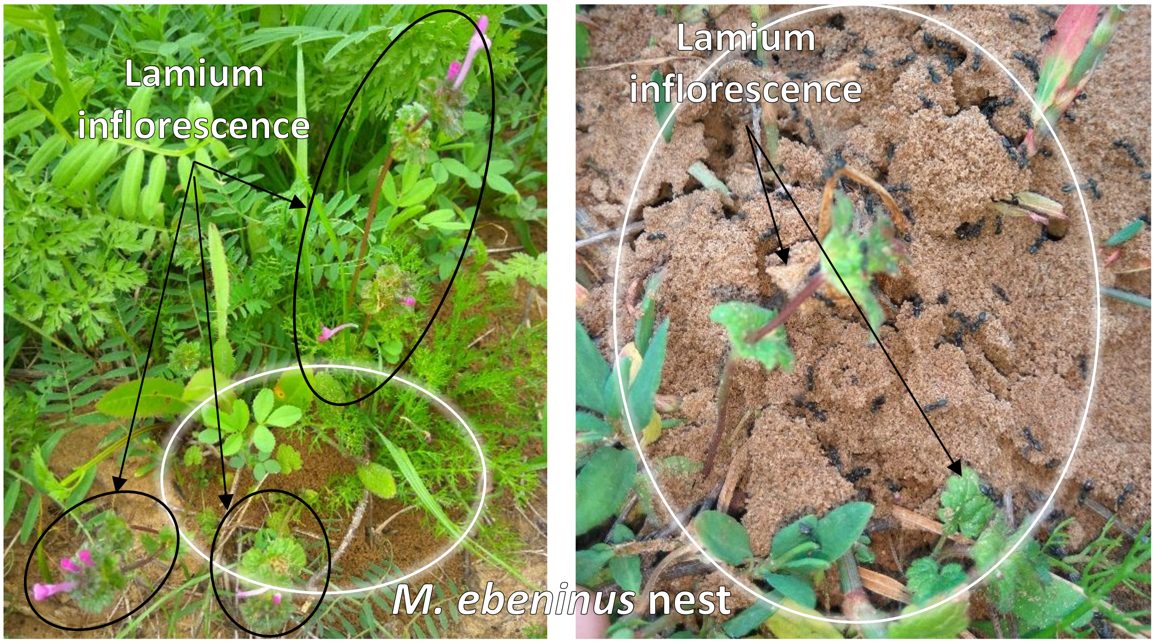

Figure S2 An *M. ebeninus* nest (white oval shapes) and the inflorescences of *L. amplexicaule* emerging through the nest area (black oval shapes and arrows). The germination of *L. amplexicaule* plants from the very nests of the dispersing ants shows that at least some of the seeds survive and germinate in great proximity to the ants.

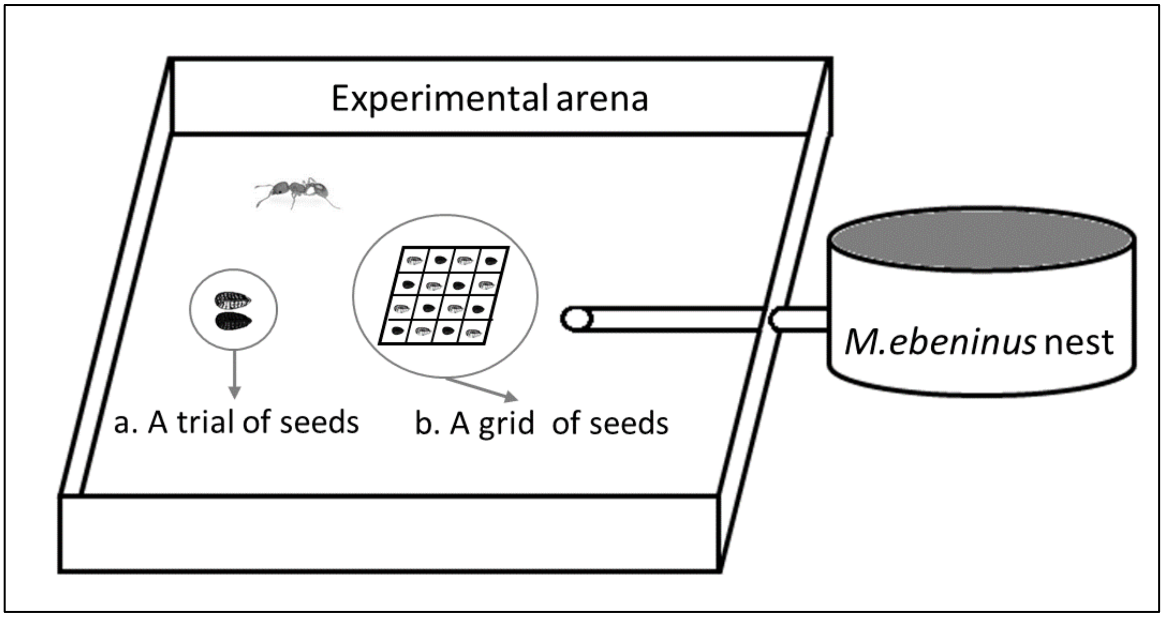

Figure S3 A scheme of the laboratorial *M. ebeninus* nest which was used for the seeds' preference test. The $1m^2$ experimental arena was connected with the ants' nest to allow the ants to pass freely between the two components. **(a)** An example of pair-based trial placed in the experimental arena (see Fig. 5). **(b)** An example of grid-based board placed in the experimental arena (see Fig. 6).

Sawing depth (cm)	Germination percent (%)	No. of germinated seeds/ Seeds sowed
**0**	4.2	3/70
**3-6**	68.5	48/70

Table S1 Germination success at different sawing depths. Seeds sowed on the soil surface germinated in significantly lower rates in comparison to seeds that were buried in the depth of 3-6 cm (Two-sample proportion test, X^2= 62.1, p<0.0001). In a previous study, it was suggested that seeds of *L. amplexicaule* can sense light and demonstrated lower germination rates when exposed to light [65]. In addition, we suggest that ants carry the *L. amplexicaule* seeds into both darkened and predation protected locations among their seed storage compartments underground. Put together, the myrmecochorous interaction may provide protection and be used as signal for the seeds for adequate germination conditions.

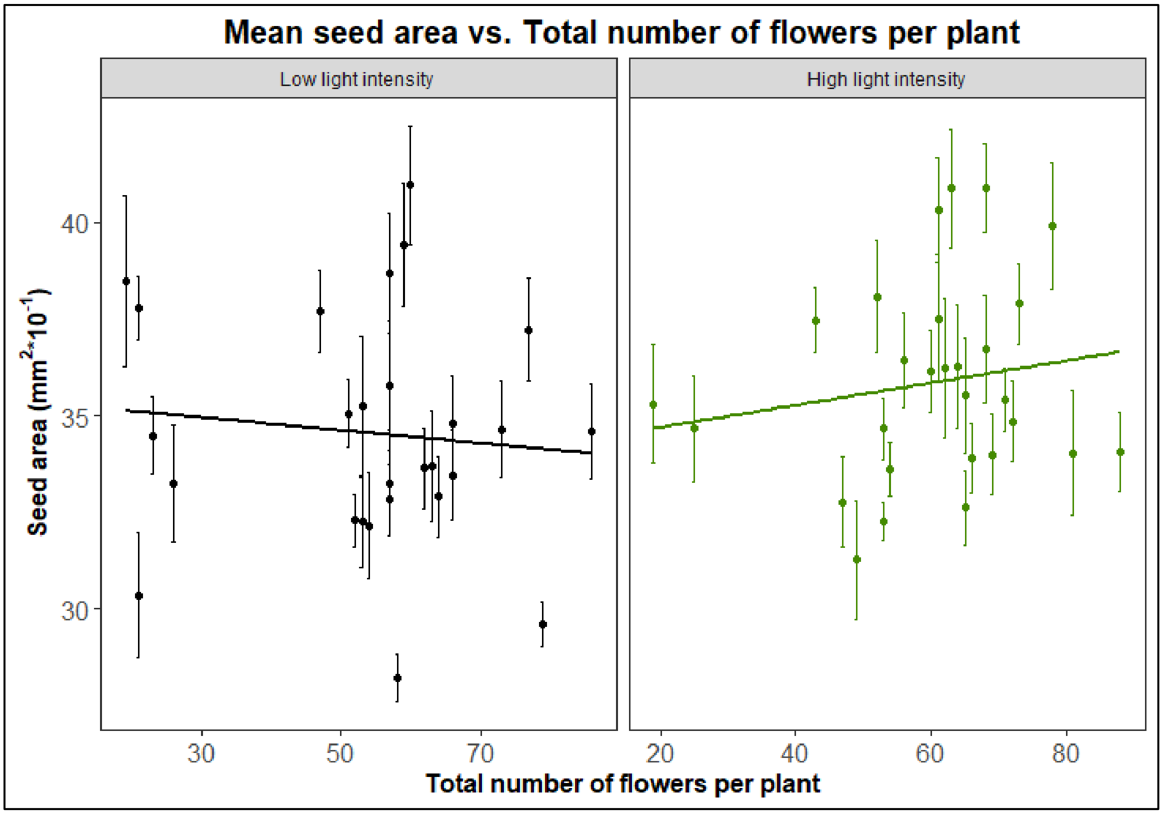

Figure S4 The area of the seed surface was not significantly correlated with the total number of flowers per plant (low light, black: t= -1.139, p-value =0.27, R^2= 0.01; high light, green: t=0.81, p-value = 0.42, R^2= 0.02; linear regression, n=30 plants for both samples).

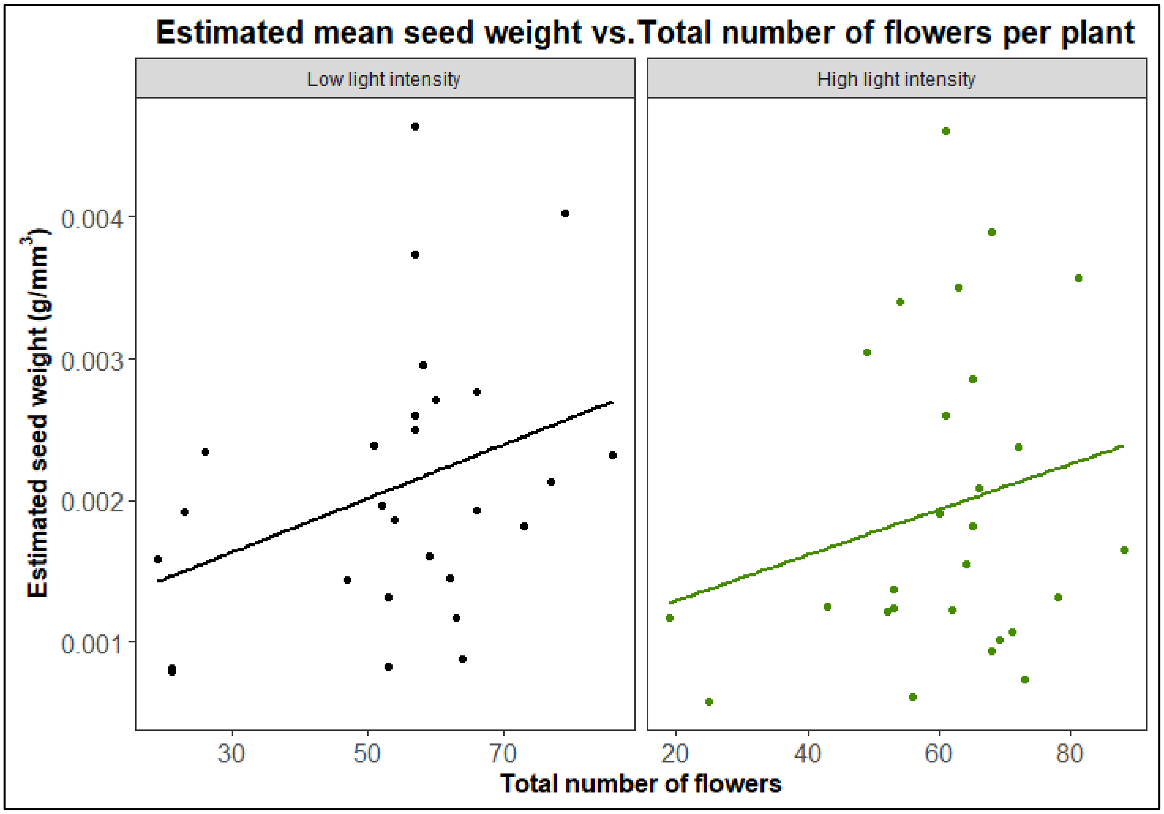

Figure S5 Seed size and the number of flowers were not significantly correlated (low light: t= 1.877, p-value =0.07, R^2= 0.09; high light: t=1.113, p-value = 0.28, R^2= 0.01; linear regression, n=30 plants for both samples). Seed size was calculated by the division of total seed set weight (in grams) by the average seed area of each plant transformed to represent volume (mm^3). The comparison between total seed set weight and the total number of flowers was omitted since it was statistically and visually similar to this analysis.

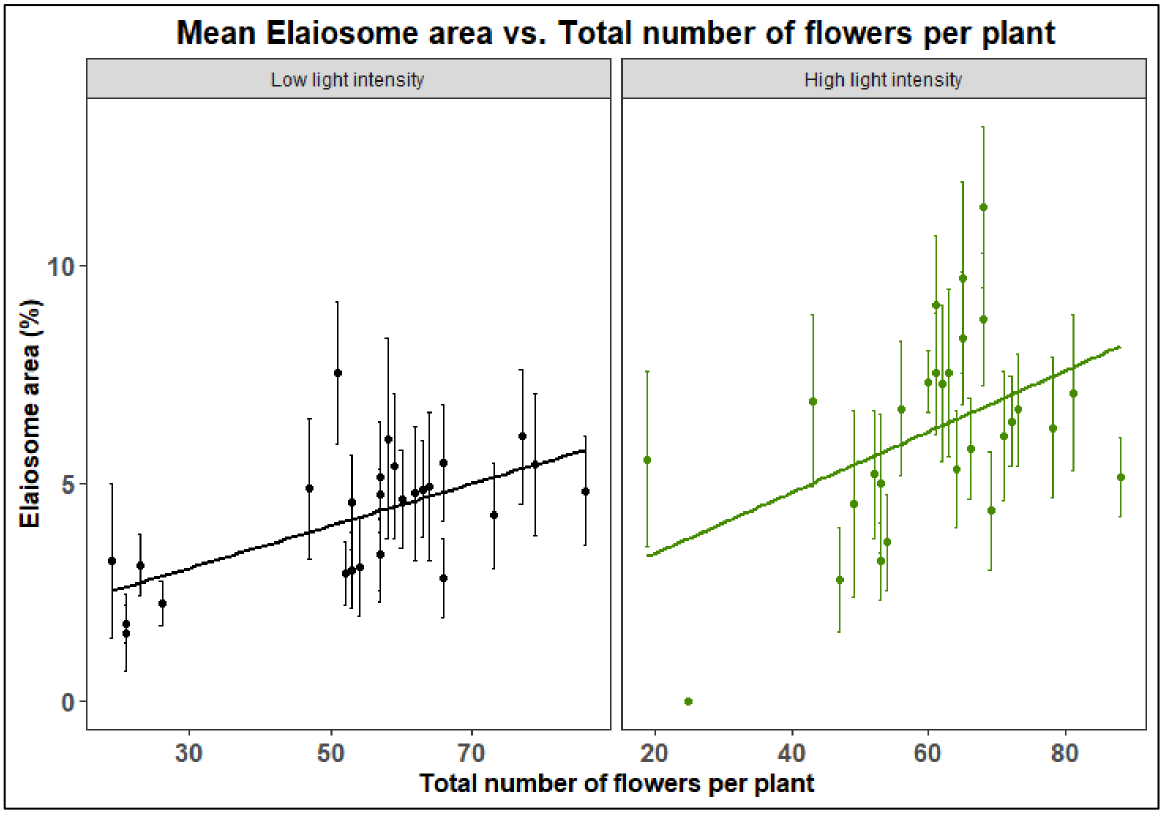

Figure S6 Mean percent of elaiosome area was positively correlated with the total number of flowers (low light: t= 4.914, p-value =4.66· 10^{-5}, R^2= 0.47; high light: t=2.603, p-value = 0.015, R^2= 0.18; linear regression, n=30 plants for both samples).

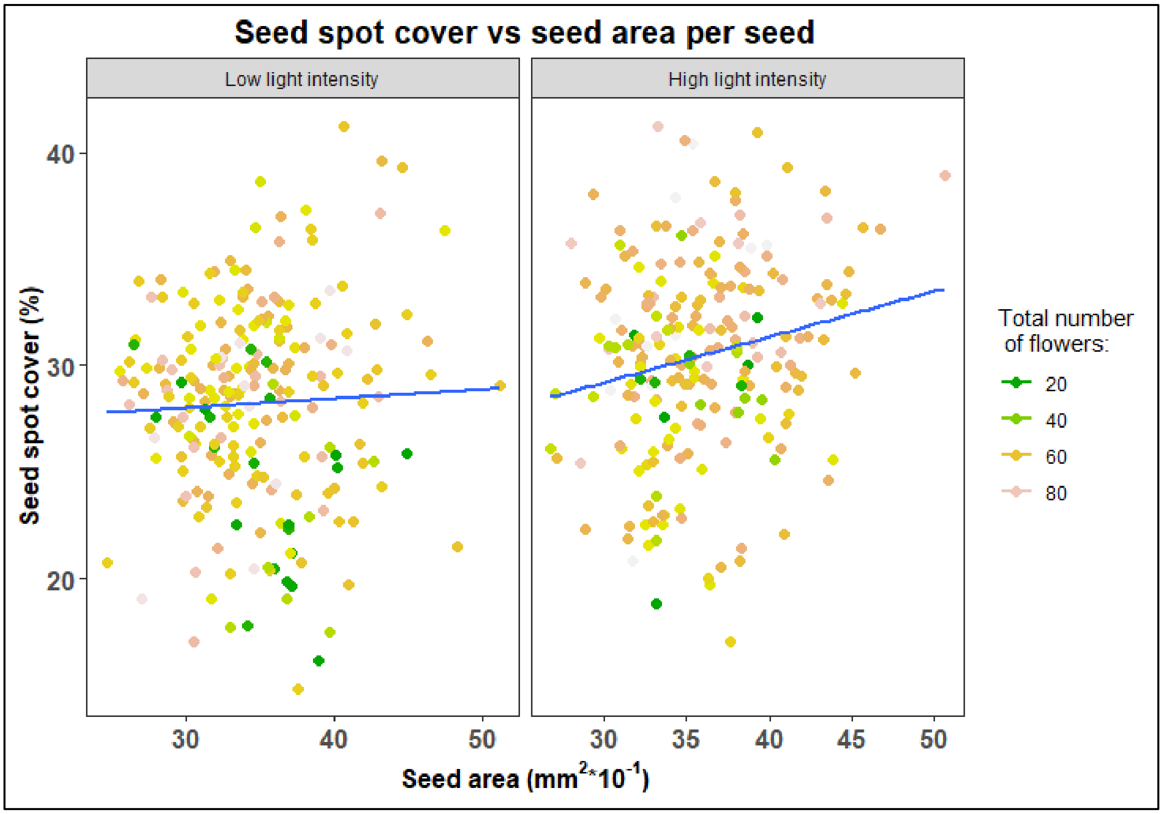

Figure S7 The percent of seed spot cover was positively correlated with the seed surface area in both the low light regime and the high light regime, showing that spotted seeds were generally slightly larger (low light: t= 3.11, p-value =$2 \cdot 10^{-3}$; high light: t=-2.84, p-value = $5 \cdot 10^{-3}$; mixed effect linear regression where a random effect for the genetic origin of each seed was added, n=220 seeds for both samples).

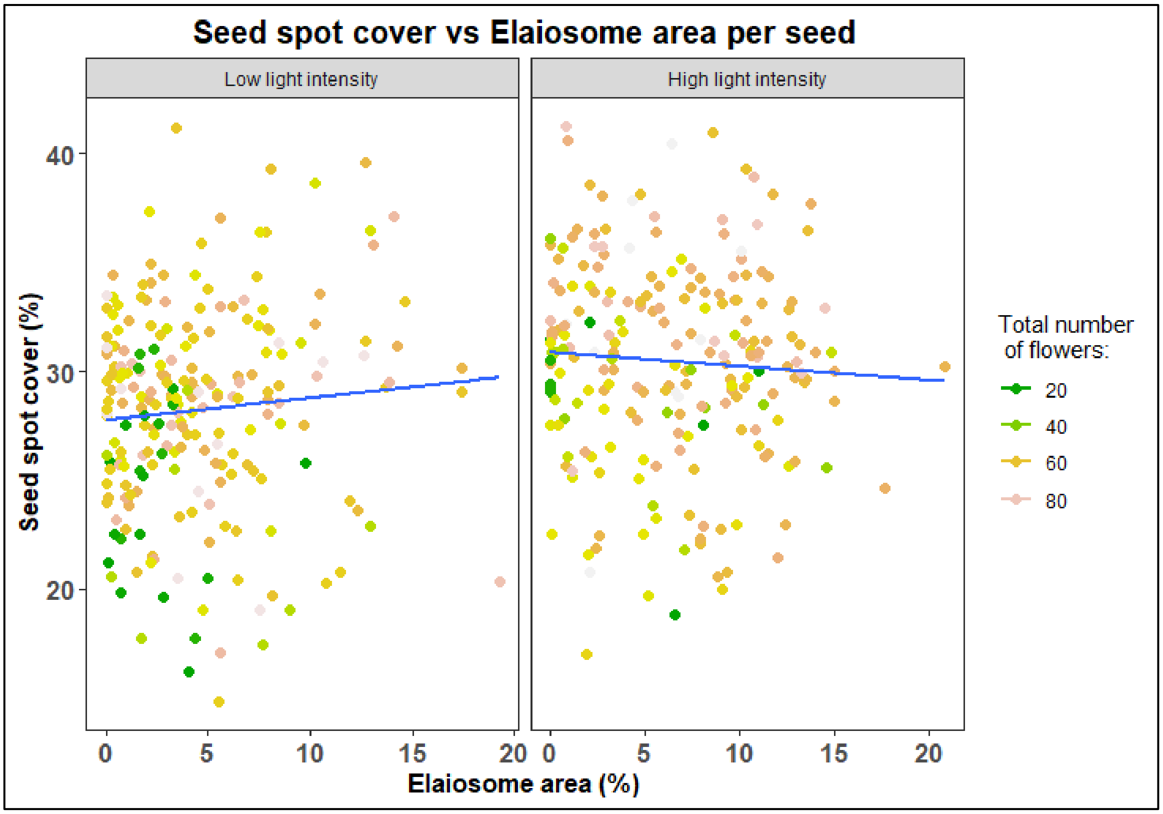

Figure S8 The percent of seed spot cover was not correlated with the percent of elaiosome area in both the low light regime and the high light regime (low light: t= 1.165, p-value =0.25; high light: t=-1.421, p-value = 0.16; mixed effect linear regression where a random effect for the genetic origin of each seed was added, n=220 seeds for both samples).

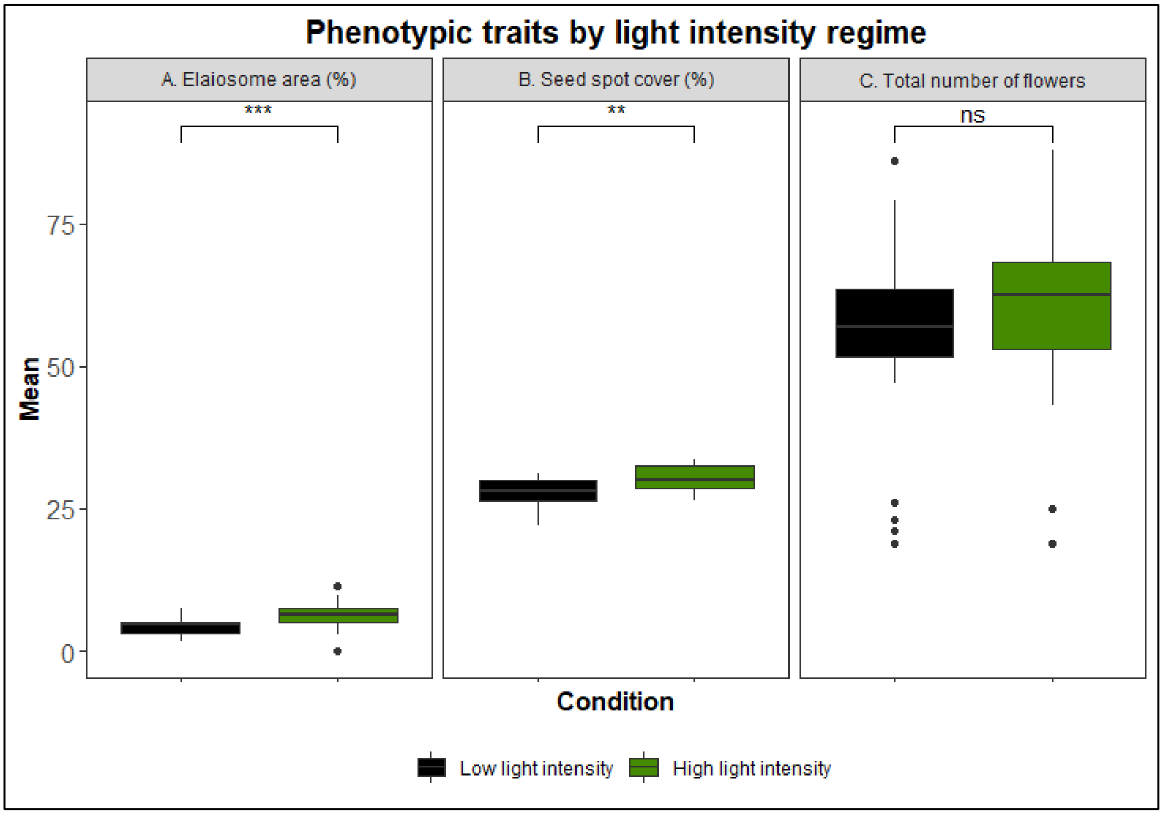

Figure S9 A comparison of different seed traits produced in low light and high light regime**:** elaiosome area was smaller in low light (plants had 6.2% and 4.2% elaiosome area of the total seed area when grown in high or low light intensities, respectively; t= 3.823, p-value =3.5· 10^{-4}; linear regression, n=30 plants for each of the samples), seed spot cover was lower in low light (plants had 30.3% and 27.9% seed spot cover when grown in high or low light intensities, respectively; t= 3.733, p-value =4.6· 10^{-4}; linear regression, n=30 plants for each of the samples) and total number of flowers not significantly different in the two regimes (plants had 60.2$\pm$ 2.8 and 54.1$\pm$3.4 flowers when grown in high or low light intensities, respectively; t= 1.373, p-value =0.18; linear regression, n=30 plants for each of the samples).

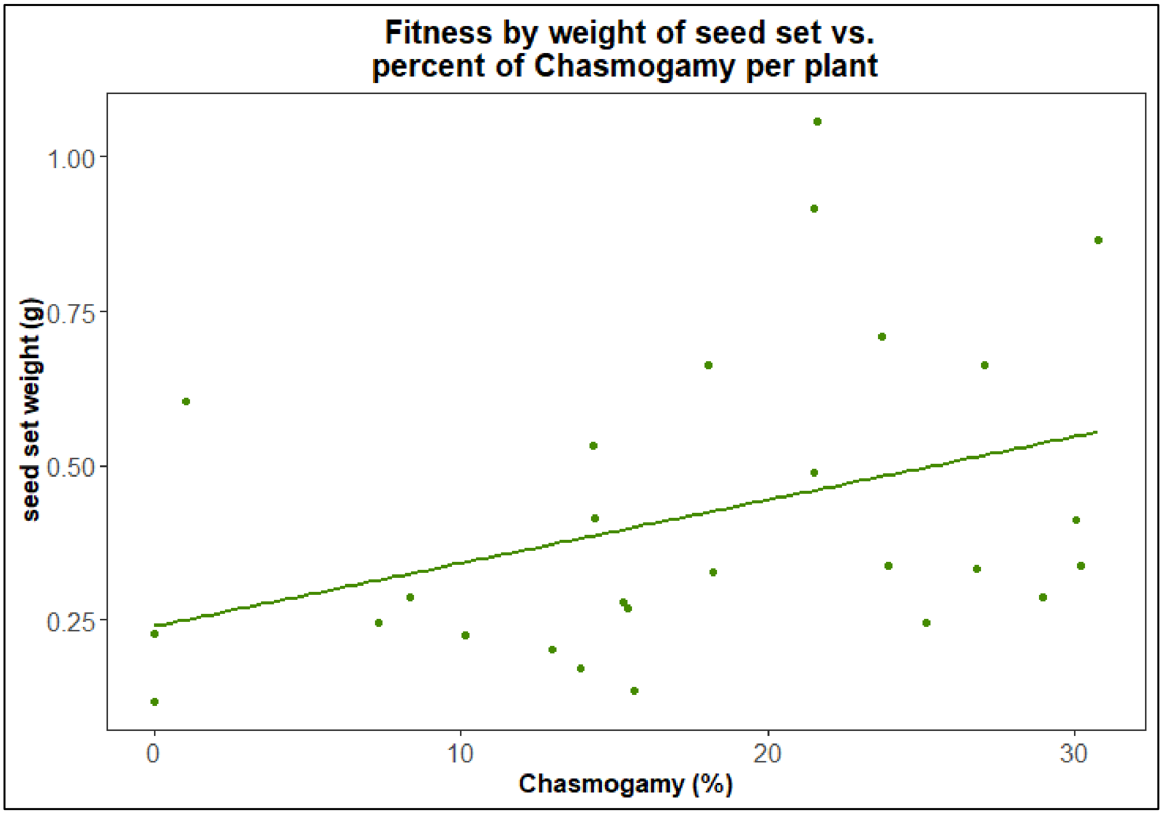

Figure S10 Seed set weight was not significantly correlated with the percent of chasmogamy per plant under high light regime, although a positive trend was observed (t= 2.02, p-value =0.054, R^2= 0.11; linear regression).

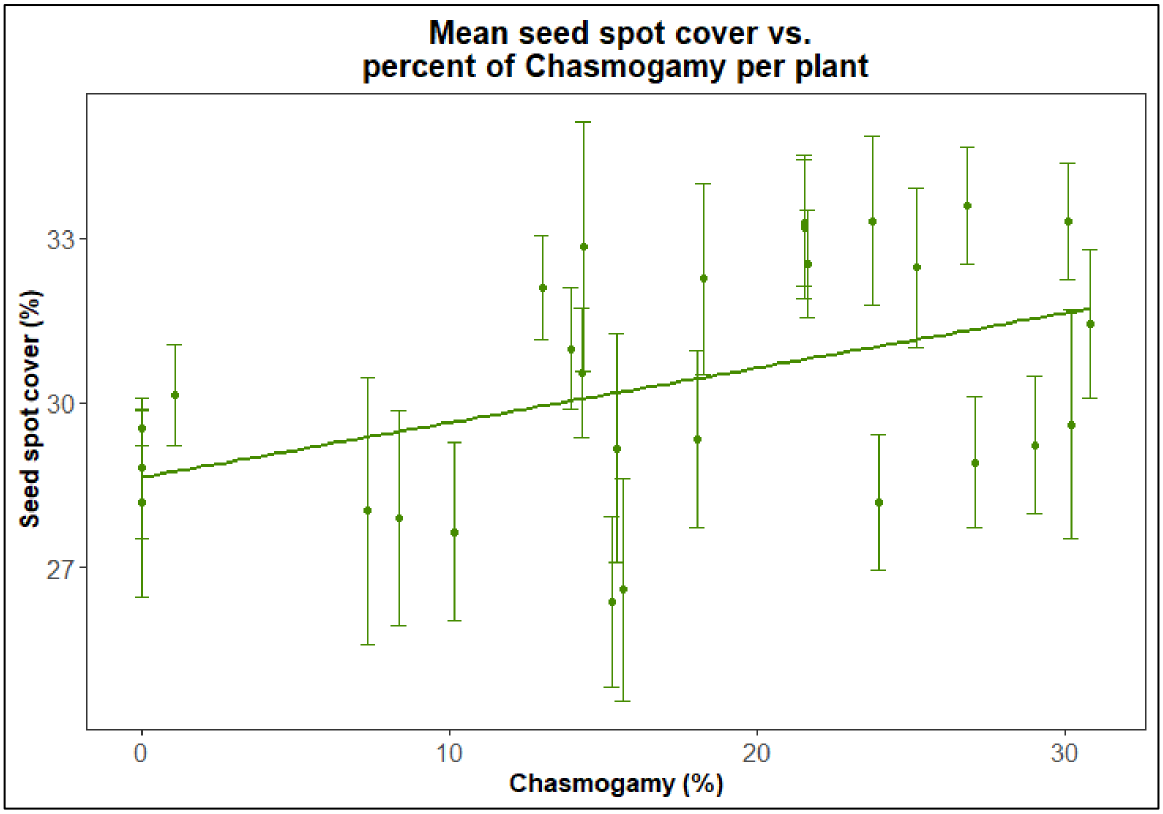

Figure S11 Seed spot cover was positively correlated with the percent of chasmogamy per plant under high light regime (t= 2.402, p-value =0.025, R^2= 0.15; linear regression).

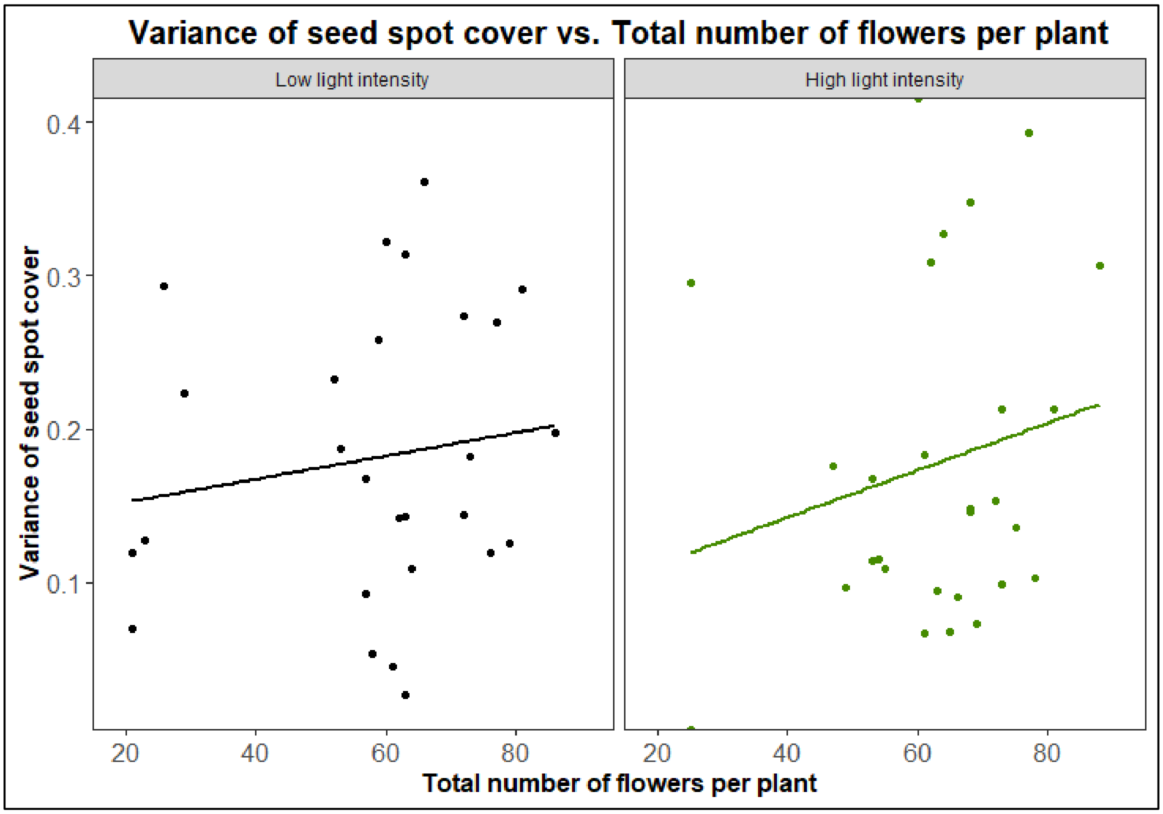

Figure S12 The within plant variance in seed spot cover was not correlated to the total number of flowers, showing that seed spot cover was not different with the fitness (low light: t= 0.283, p-value =0.78, R^2= 0.01; high light: t=0.322, p-value = 0.75, R^2= 0.01; linear regression, n=30 plants for both samples).
